# Interfering with the high-affinity interaction between wheat amylase trypsin inhibitor CM3 and toll-like receptor 4: *in silico* and biosensor-based studies

**DOI:** 10.1038/s41598-017-13709-1

**Published:** 2017-10-13

**Authors:** Massimiliano Cuccioloni, Matteo Mozzicafreddo, Laura Bonfili, Valentina Cecarini, Mara Giangrossi, Maurizio Falconi, Shin-Ichiroh Saitoh, Anna Maria Eleuteri, Mauro Angeletti

**Affiliations:** 1 School of Biosciences and Biotechnology, University of Camerino, 62032 Camerino, Italy; 2 Division of Innate Immunity, Department of Microbiology and Immunology, The University of Tokyo, 4-6-1 Shirokanedai, Minatoku, Tokyo, 108 8639 Japan

## Abstract

Wheat amylase/trypsin bi-functional inhibitors (ATIs) are protein stimulators of innate immune response, with a recently established role in promoting both gastrointestinal and extra-gastrointestinal inflammatory syndromes. These proteins have been reported to trigger downstream intestinal inflammation upon activation of TLR4, a member of the Toll-like family of proteins that activates signalling pathways and induces the expression of immune and pro-inflammatory genes. In this study, we demonstrated the ability of ATI to directly interact with TLR4 with nanomolar affinity, and we kinetically and structurally characterized the interaction between these macromolecules by means of a concerted approach based on surface plasmon resonance binding analyses and computational studies. On the strength of these results, we designed an oligopeptide capable of preventing the formation of the complex between ATI and the receptor.

## Introduction

In the last decades, the implementation of novel agricultural practices contributed positively to the decrease of costs associated with large-scale production of wheat-based food. Consequently, the higher consumption of breads and pastas caused a predictable increase in hypersensitization to wheat. The most common of these disorders include baker’s asthma^1^, and immune reactions to wheat ingestion, such as celiac disease (CD), wheat allergy (WA), and non-celiac gluten/wheat sensitivity (NCGS or NCWS)^2–5^.

CD is triggered by gluten peptides that induce the adaptive immune response in predisposed individuals, resulting in the activation of T-cells^6,7^, whereas IgE antibodies are induced by wheat proteins in WA, eventually stimulating the release of immune mediators^8^.

On the other hand, NCGS is associated with innate immune activation, which is likely stimulated by wheat proteins^9,10^. NCGS presents also extra-intestinal symptoms^11^, such as confusion and headache, chronic fatigue, joint/muscle pain, and the exacerbation of pre-existing neurological, psychiatric, or (auto-)immune diseases^4,12,13^.

Based on their structural, chemical and physical properties^10^, wheat proteins are generally categorized as albumins and globulins (15% of total protein content), and gluten (85% of total protein content). Specifically, gluten consists of a complex mixture of monomeric gliadins and polymeric glutenins, whereas albumins and globulins comprise several families of proteins, such as the α-amylase/trypsin inhibitors (ATIs), β-amylases, peroxidases, lipid transfer proteins, and serine proteases inhibitors^10^. In the quest to identify wheat components effectively responsible for the initiation of innate immune response, ATIs were demonstrated as potent activators of myeloid cells. Specifically, ATIs directly engage TLR4–MD2–CD14 complex and activate both nuclear factor kappa B and interferon responsive factor 3 pathways, resulting in the up-regulation of maturation markers and the release of proinflammatory innate cytokines^14^. The centrality of TLR4 system was further confirmed, as animal models deficient in TLR4 were protected from the intestinal and systemic immune responses upon oral challenge with ATIs^15^.

Compared to other protein constituents, ATIs represent a minor, but still significant part of total wheat proteins (2–4%)^16^, on average, an adult person being exposed up to 1 g of ATIs *per* day: in fact, ATIs are present and even enriched in commercial wheat-based food^17^, and can escape proteolytic digestion by pepsin and trypsin, preserving the TLR4-activating ability after intestinal transit upon oral ingestion^18^.

Structurally, wheat ATIs belong to a group of hydrolase-resistant proteins stabilized by inter-molecular disulfide bonds^19^, and with high secondary structural homology^15^. They can be further divided into three sub-groups constituted by monomeric and (non-covalently linked) dimeric and tetrameric forms^20^. ATIs are found in the endosperm of plant seeds, where they represent part of the natural defence against parasites and insects, as well as regulatory molecules of starch metabolism during seed development and germination^21,22^. Plants other than wheat, such as rye and barley also contain similar bi-functional inhibitors, but show only minimal or absent TLR4-activating activity^15^.

Due to the *in vivo* TLR4 stimulatory activity and resistance to gastrointestinal proteolysis^15^, this latter being attributable to the potent inhibitory activity toward diverse hydrolases^23^, ATIs may exert a pathogenic role in inflammatory, metabolic and autoimmune diseases and in NCGS^11,24,25^.

On the strength of the interplay between ATIs and TLR4, in this study we used the *IAsys plus* system to explore the kinetics of the interaction between a representative member of wheat ATI family, namely CM3, and human TLR4. In addition, we performed molecular docking studies to predict the structural basis of ATI-TLR4 complex, evaluating the most probable binding sites and interaction forces, and identifying the residues at the binding interface. Interestingly, besides revealing univocally a high-affinity interaction between the two macromolecules, the results of the concerted computational and binding studies led to design an oligopeptide constituting part of the discontinuous ATI binding interface with TLR4, which was able to prevent the interaction between the two macromolecules.

## Results

### Biosensor binding studies

**Figure 1:**
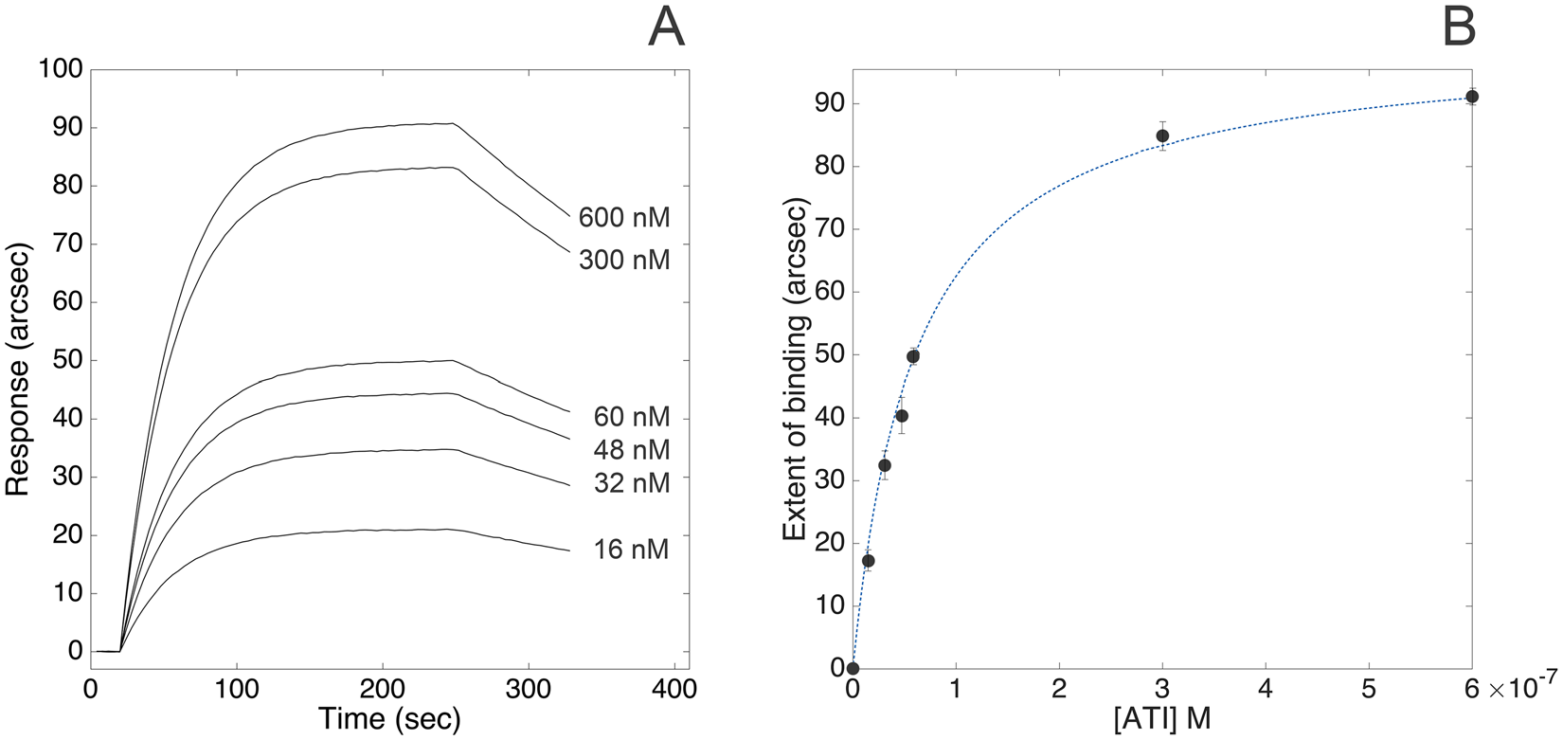
Binding of ATI to surface-blocked TLR4. Superimposition of sensorgrams obtained at increasing concentrations of the bi-functional inhibitor (Panel A). Extent of binding vs concentration plot (Panel B).

Carboxylate cuvettes were selected to covalently immobilize TLR4 *via* primary amines. The very high stability of the resulting biosensing layer combined with the low instrumental short-term noise (less than 1 arcsec) granted an accurate determination of both kinetic and equilibrium parameters of the interaction between human TLR4 and wheat ATI CM3 under different pH and ionic strength conditions. The superimposition of representative association kinetics obtained upon binding of different concentrations of ATI to TLR4 (PBS pH 7.4, supplemented with 140 mM NaCl) is reported in Fig. 1, Panel A. Raw data were routinely accumulated over a 6-min interval. Association and dissociation curves were fitted to both mono- and bi-exponential models: since bi-exponential models did not significantly improve the quality of fits, as judged by standard F-test, 95% confidence, the mono-exponential models (Eqq. 3 and 5, see Method section) were used throughout to analyse the data.

This experimental approach evidenced the high-affinity interaction between soluble ATI and surface-blocked receptor (*K*_*D, kin*_ = (6.1 ± 1.7) × 10^−8^ M, as calculated from the ratio of kinetic parameters derived from Eq. 2). Both fast association (*k*_*ass*_ = (4.1 ± 0.6) × 10^4^ M^−1^ s^−1^) and slow dissociation (*k*_*diss*_ = (2.5 ± 0.6) × 10^−3^ s^−1^) phases significantly contributed to the stabilization of the complex.

The binding response at equilibrium (extent of binding) was calculated for each time course, with fully comparable equilibrium dissociation constant value (*K*_*D, ext*_ = (3.6 ± 1.5) × 10^−8^ M, as directly calculated from Eq. 4) with respect to values derived from kinetic analyses (Fig. 1, Panel B).

Binding stoichiometry was determined to be 1:1, as calculated from the molar ratio between surface-blocked TLR4 (59 μM, directly estimated from the biosensor response upon immobilization) and soluble ATI (60 μM, derived from the extent of binding at saturating concentration of the bi-functional inhibitor).

### Effect of pH and ionic strength

**Figure 2:**
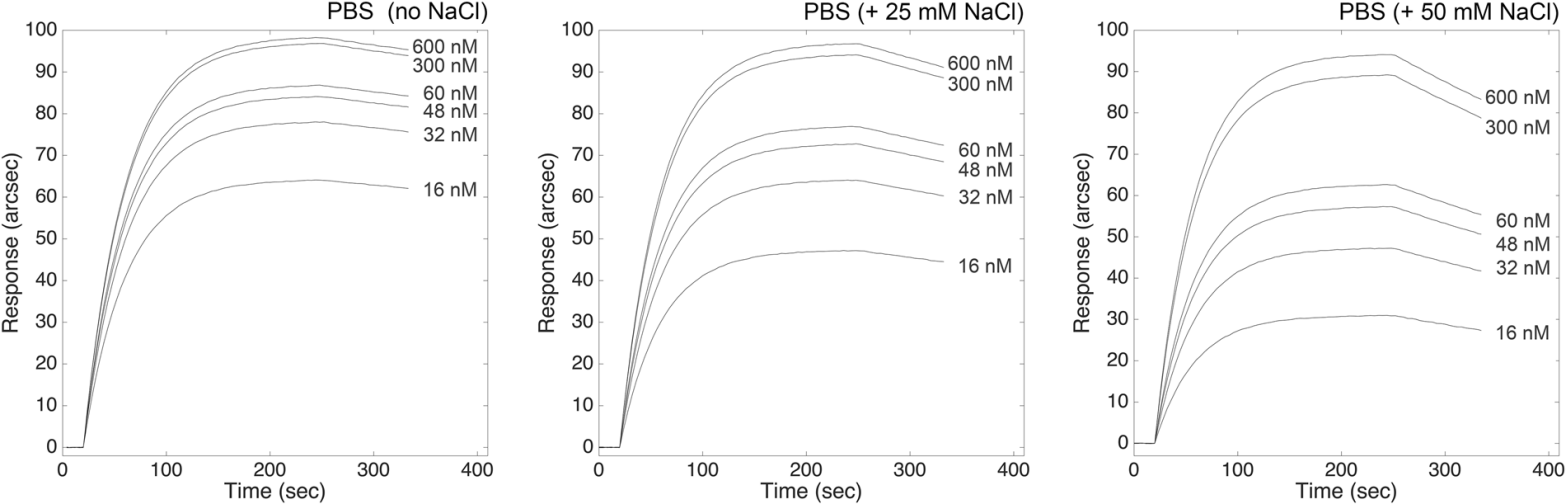
Effect of ionic strength lowering on the interaction between ATI and surface-blocked TLR4. Superimposition of sensorgrams obtained at increasing concentrations of the bi-functional inhibitor, each at different ionic strength conditions.

We investigated the influence of pH and ionic strength on the kinetic and equilibrium parameters of the binding between ATI and TLR4 (all parameters are summarized in Table 1). At lower salt concentrations, negatively-charged TLR4 bound with higher affinity to ATI, exclusively due to the higher kinetic stability of the ATI-TLR4 complex (lower value of kinetic dissociation constant). Both complex affinity and kinetic stability progressively decreased with increasing ionic strength, with a final 25-fold loss in complex stability (as also evident from the progressive increase in the slope of the dissociation phases with NaCl concentration, Fig. 2). For this reason, we used PBS supplemented with 200 mM NaCl instead of using HCl 10 mM for the regeneration step (this procedure being likely to cause denaturation of the macromolecule blocked on the cuvette surface). Conversely, kinetics of association were not affected by salt concentration, the changes in *k*_*ass*_ values being negligible within experimental errors.

**Table 1:** Effect of ionic strength on kinetic and equilibrium parameters of the interaction between wheat ATI and TLR4.

Ionic strength conditions	k_ass_ (M^−1^s^−1^)	k_diss_ (s^−1^)	K_D_ (M)
20 mM PBS (no NaCl)	43000 ± 2000	0.0001 ± 0.00005	(2.3 ± 1.2) × 10^−9^
20 mM PBS (25 mM NaCl)	39000 ± 4000	0.0004 ± 0.0002	(1.0 ± 0.5) × 10^−8^
20 mM PBS (50 mM NaCl)	40000 ± 2500	0.0007 ± 0.0001	(1.8 ± 0.3) × 10^−8^
20 mM PBS (140 mM NaCl)	41000 ± 6000	0.0025 ± 0.0006	(6.1 ± 1.7) × 10^−8^

Neither kinetic nor equilibrium parameters showed significant dependence from pH in the range 6–8 (data not shown).

### Electrostatic potential maps

ATI-TLR4 complex was mainly stabilized by electrostatic interactions. In fact, TLR4 revealed a large, global negative charge (for pH values higher than 5), whereas ATI, presenting both positively and negatively charged surfaces, was supposed to act as a protein dipole (See Supplementary Information). These results were in good agreement with the effects induced by changes in the dielectric constant of the buffer solution on complex stability (high ionic strength conditions strongly destabilized ATI-TLR4 complex).

### Docking analysis of ATI to TLR4

**Figure 3:**
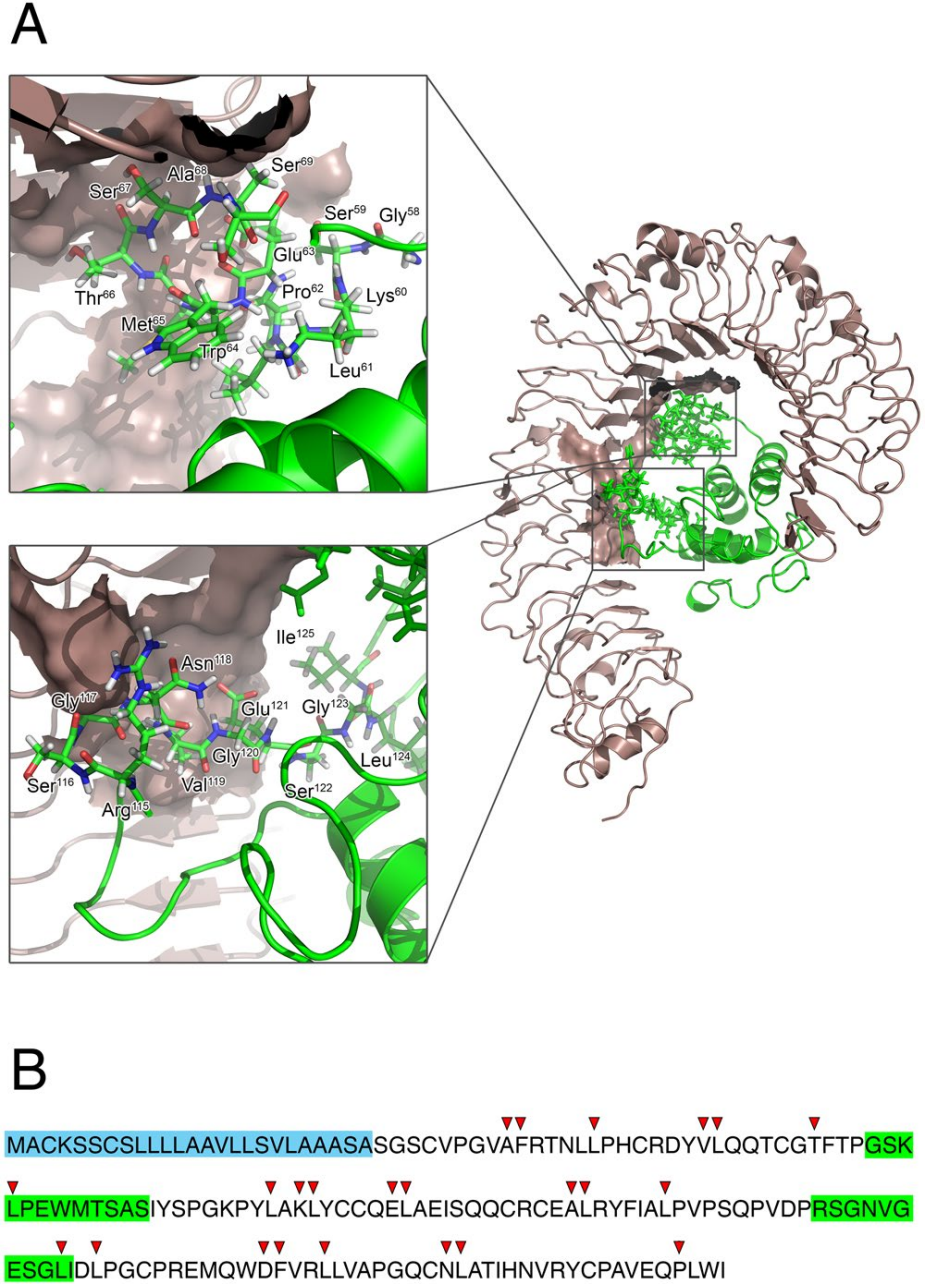
Three-dimensional representation of the molecular docking of homology modelled wheat ATI CM3 (green ribbon) onto human TLR4-MD2 complex (grey ribbon). For better clarity of ATI-TLR4 complex visualization, MD2 molecule was removed after docking procedure. Black box highlights the oligopeptides constituting the discontinuous ATI binding interface. Oligopeptides are visualized as sticks (Panel A). Predicted cleavage sites by pepsin on wheat ATI CM3 (Panel B). The oligopeptides constituting the discontinuous ATI binding interface are highlighted in green. Residues constituting signal peptide (excluded from docking procedure) are highlighted in light blue.

Docking studies between the fold-recognition model of wheat ATI CM3 (see Experimental Section for details) and the X-ray crystal structure of the human TLR4-MD2 complex disclosed new insights regarding both the nature of the interaction and the binding geometry of the complex. The inner β-strand rich region of human TLR4 was calculated to be the most likely to accommodate the ATI molecule (Fig. 3, Panel A), with a predicted equilibrium dissociation constant of 9 × 10^−8^ M, in excellent agreement with the experimental results. Predictive ATI-TLR4 binding interface regions are shown in Fig. 3, Panel A, and amino acid sequences are presented in Fig. 3, Panel B.

### Binding of ATI upon enzymatic digestion

**Figure 4:**
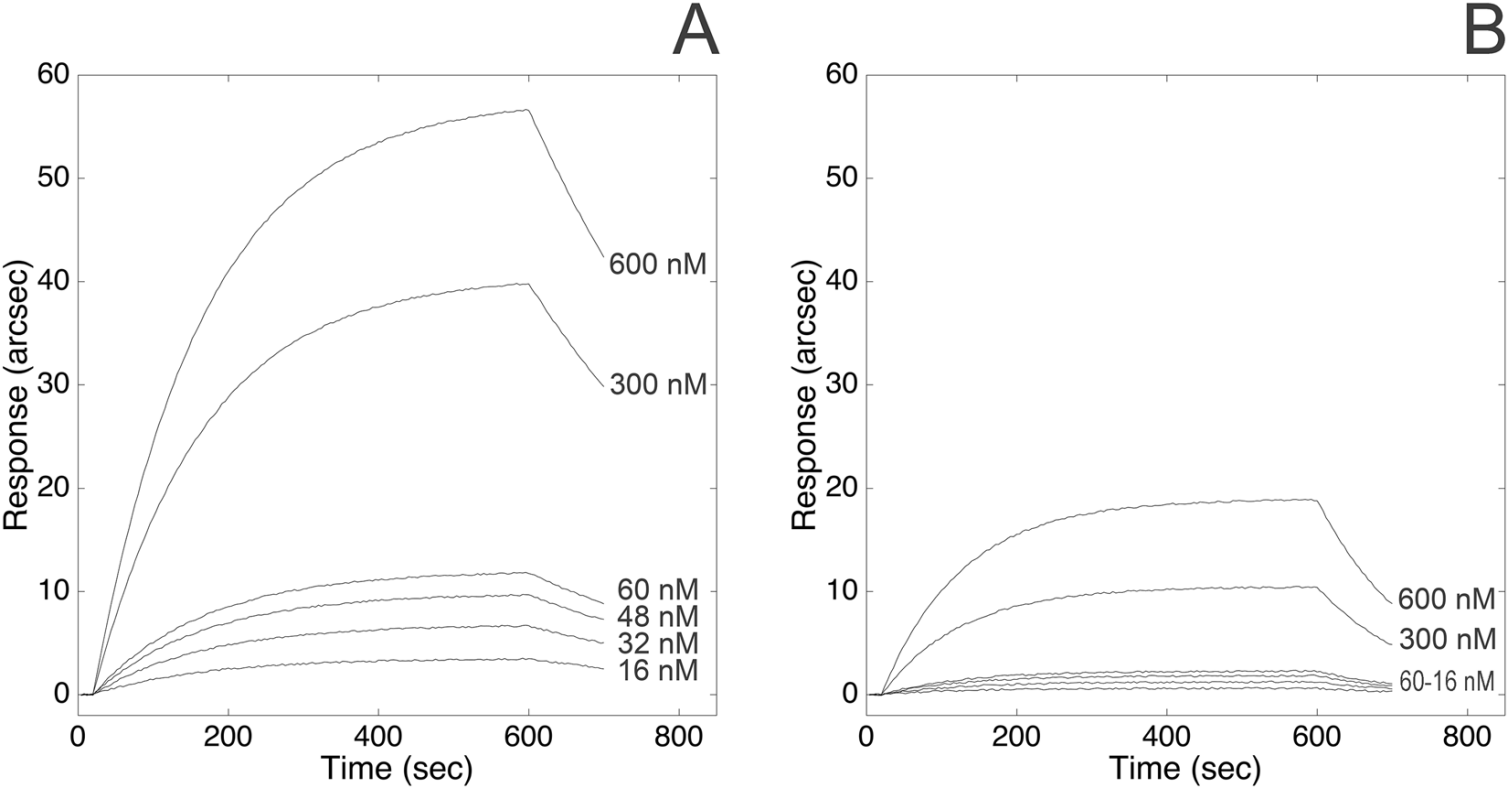
Binding of pepsin-digested ATI to surface-blocked TLR4. Comparison of responses obtained at increasing concentrations of the ATI digested under non-reducing (Panel A) and reducing conditions (Panel B).

Based on the results of docking and predictive protease cleavage sites studies, a minor portion of the discontinuous ATI binding interface was unaffected by pepsin treatment. The binding to TLR4 was tested also upon digestion of ATI with pepsin under both reducing and non-reducing conditions (Fig. 4), since native wheat ATIs are strongly stabilized by four intramolecular disulfide bonds, which confer resistance to enzymatic hydrolysis. As evident from the comparison of equilibrium dissociation constants, ATI largely preserved its binding ability upon digestion under non-reducing conditions (native ATI is strongly stabilized by four intramolecular disulfide bonds, which confer resistance to enzymatic hydrolysis), with a nearly 8-fold decrease in *K*_*D*_, exclusively attributable to the less favourable recognition phase (Table 2). Conversely, the digestion of ATI under reducing and acetylating conditions with 2-mercaptoethanol and iodoacetamide yielded a product still capable of binding to TLR4. This product displayed further lower affinity for TLR4 (nearly 43-fold lower than undigested ATI), both association and dissociation rates being significantly affected by the treatment.

**Table 2:** Comparison of kinetic and equilibrium parameters of the interaction between TLR4 and both ATI (either non-digested and digested by pepsin) and the oligopeptide constituting part of the ATI binding interface.

*Complex*	*k*_*ass*_ (M^−1^ s^−1^)	*k*_*diss*_ (s^−1^)	*K*_*D*_ (M)
TLR4-ATI	41000 ± 6000	0.0025 ± 0.0006	(6.1 ± 1.7) × 10^−8^
TLR4-ATI_nonred_dig_	6000 ± 400	0.0029 ± 0.0004	(4.8 ± 0.7) × 10^−7^
TLR4-ATI_red_dig_	3000 ± 350	0.0077 ± 0.0010	(2.6 ± 0.5) × 10^−6^
TLR4-*RSGNVGESGLI*	3620 ± 280	0.0050 ± 0.0012	(1.4 ± 0.4) × 10^−6^

### Competitive binding of a short ATI-derived peptide to TLR4

The results of computational analyses led to the identification of an oligopeptide (amino acid sequence: *RSGNVGESGLI*) constituting a significant portion of the discontinuous ATI binding interface to TLR4. This oligopeptide can largely escape pepsin digestion (the analysis of predictive protease cleavage sites with PeptideCutter^26^ evidenced the hydrolysis of only the last residue of the motif bearing the sequence of interest, as shown in Fig. 4), and (most importantly) is likely to antagonize ATI action by interfering with the formation of the ATI-TLR4 complex. In fact, according to molecular docking analysis with Autodock Vina, the peptide accommodated in a region of TLR4 constituting part of ATI binding interface: more specifically, Arg115 residue at the amino-terminal end of the *RSGNVGESGLI* peptide was predicted to interact with Asp379 present in TLR4, while Val119 of the oligopeptide is in contact with Tyr403 and Tyr451 residues of TLR4 (see Supplemental Material).

**Figure 5:**
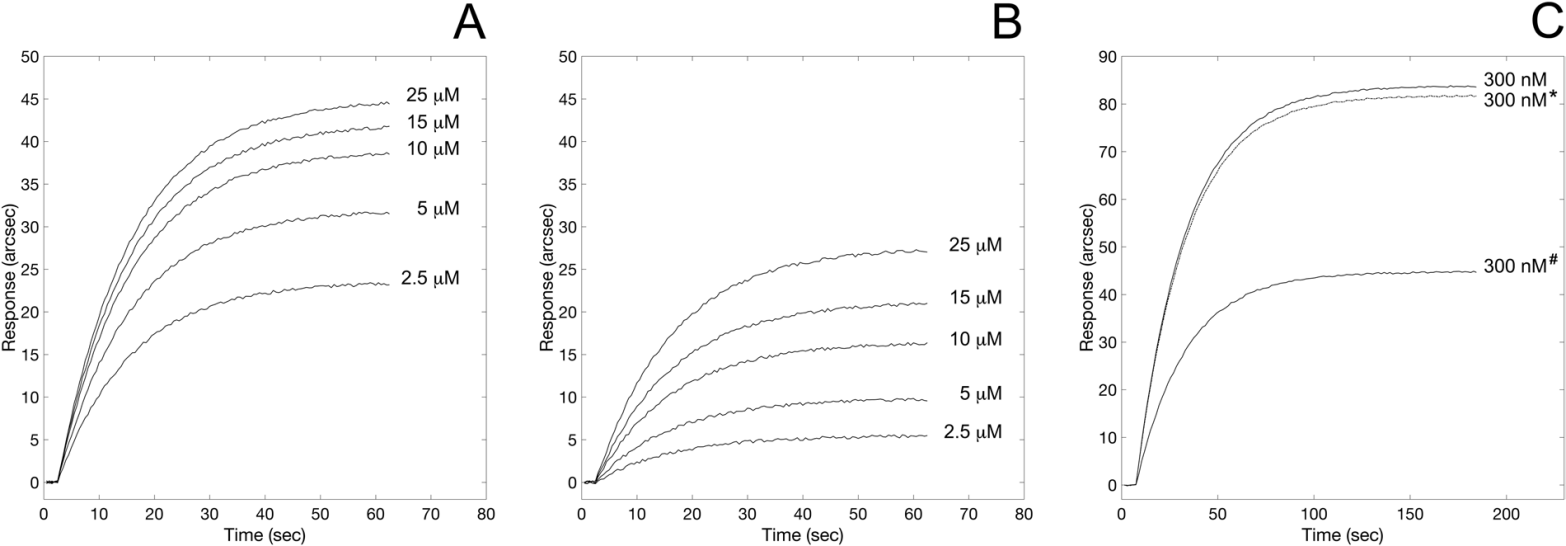
Binding of ATI-derived oligopeptides to surface-blocked TLR4. Superimposition of sensorgrams obtained at increasing concentrations of the straight peptide (*RSGNVGESGLI*, Panel A), and of the scrambled counterpart (*SGIVLSGGRNE*, Panel B). Competitive binding to TLR4 (Panel C). Comparison of binding of ATI to free surface-blocked TLR4, and upon pre-saturation of the receptor with *RSGNVGESGLI* (^#^-marked curve) and with *SGIVLSGGRNE* (*-marked curve).

According to this premise, first we tested the ability of the oligopeptide to effectively bind to TLR4 using the same biosensor-based assay described above. The oligopeptide exhibited specific binding to the extracellular domain of TLR4 (Fig. 5, Panel A) with a 1:1 binding stoichiometry, (calculated as described above for the ATI-TLR4 complex). The equilibrium dissociation constant (*K*_*D,StrP*_ = (1.4 ± 0.4) × 10^−6^ M) was compatible with the value measured upon binding between TLR4 and pepsin-digested ATI under reducing/alkylating conditions. Most interestingly, upon pre-saturation of TLR4 with 100 μM *RSGNVGESGLI*, the binding of ATI to the receptor was largely prevented, as evident from the corresponding 50% reduction in the maximal response at equilibrium (Fig. 5, Panel C). To address the specificity of the peptide–receptor interaction, a scrambled version of the peptide (amino acid sequence: *SGIVLSGGNRE*, generated using RandSeq tool^27^) was tested both for its binding ability to TLR4 and for competitive activity to ATI: the scrambled counterpart showed no competitive activity (Fig. 5, Panel C), still being capable of binding to the receptor although with lower affinity (*K*_*D,ScrP*_ = 9.9 × 10^−6^ M) (Fig. 5, Panel B).

## Discussions

Toll-like receptors are ubiquitous in immune cells^28,29^, in which they mediate the stimulation of the innate response and enhance adaptive immunity against pathogens^30^. In this context, their activation may result in the onset of autoimmune, chronic inflammatory and infectious diseases^31^. Specifically, nutritional ATI proteins from wheat were reported to activate the TLR4–MD2–CD14 complex^15^ according to a lipopolysaccharide-like mechanism, and elicit strong innate immune effects *in vitro* and *in vivo*, with consequent profound implications both in gastrointestinal inflammatory disorders (celiac disease, gluten sensitivity, irritable bowel syndrome, inflammatory bowel disease), and in non-intestinal inflammation.

According to a concerted approach based on surface plasmon resonance biosensor and molecular docking methods, we explored the interaction between a representative member of the structurally conserved ATI family, namely CM3, and human TLR4, demonstrating ATI ability to directly target the receptor. The resulting 1:1 ATI-TLR4 complex was characterized by *K*_*D*_ in the nanomolar range. ATI binding to TLR4 occurred with fully comparable affinity to that of TLR4 physiological partner MD2^32^, and similar to other ATI targets, such as digestive serine proteases and amylases^23^. Additionally, wheat ATI CM3 preserved part of the native TLR4-binding ability upon *in vitro* enzymatic digestion, confirming the role of intramolecular disulphide bonds as key determinants of wheat ATI capacity to survive gastro-intestinal transit and trigger TLR4 signalling^25^.

ATI-TLR4 complex was mainly stabilized by non-covalent electrostatic interactions, and changes in ionic strength significantly altered the dissociation rate and the stability of the complex. Specifically, *k*_*diss*_ and *K*_*D*_ values increased nearly by a 25-fold factor in the presence of NaCl (up to 140 mM), as determined by analogous biosensor-based binding assays replicated under different ionic strength conditions, and in agreement with the results of the computational mapping of electrostatic potentials (see Fig. 2 and Supplemental Material).

Consistently with these experimental evidences, computational analysis predicted ATI molecule to favourably accommodate within the β-strand loop of TLR4, in a binding region distinct from both MD2 and TLR4 self-dimerization interfaces^33^. The full agreement between computational and experimentally determined equilibrium dissociation constants confirmed the quality of the predictive model.

Other works exploited the minimal binding region between TLR4 and a number of physiological binders thereof (MD2^34^, MyD88^35^, and TRAM^36^) to prevent complex formation and inflammatory downstream effects. Analogously, the mapping of the binding interfaces of TLR4-ATI model (Fig. 3) was pivotal in guiding the design and the synthesis of an antagonist 11-mer oligopeptide (largely hydrolase-resistant, as predicted by sequence analysis). Preliminary docking studies predicted the binding interface between the 11-mer and TLR to be perfectly superimposable to that of ATI (see Supplemental Material); when tested for binding to TLR4, the oligopeptide specifically bound to the receptor with partly preserved binding affinity of the parent ATI molecule, but most interestingly successfully prevented the formation of the ATI-TLR4 complex in a biosensor-based competitive binding assay. Based on these promising results, we reasonably believe that this oligopeptide could inhibit the activation of ATI-induced inflammatory cascade, consistently with previous studies reporting the abrogation of IL-1β production upon treatment with ATI digested under reducing and alkylating conditions^37^.

In conclusion, our findings may have physiological and pharmacological implications not only for celiac disease and non-celiac gluten sensitivity, but also for other gastrointestinal inflammatory disorders. Furthermore, although demanding further customization (at this stage, the nearly 30-fold lower affinity and slower association kinetics with respect to ATI would require a large excess of the oligopeptide to interfere with the interaction with TLR4) the *RSGNVGESGLI* peptide can be used as the starting point for the rational design of ligands able to specifically block the interaction between TLR4 and its activators. Further studies are currently in progress.

## Methods

### Biosensor device

Binding experiments were performed on an evanescent wave/resonant mirror^38^ optical biosensor (IAsys plus - Affinity Sensors Ltd, Cambridge, UK), equipped with dual-well carboxylate cuvettes (NeoSensors, Ltd., UK). A working volume of 80 μL was used throughout, and the temperature was set at 37 °C. The possible influence of mass transport on the determination of kinetic parameters^39^ was considered and reduced by setting the stirrer rate to 95%.

### Preparation of TLR4 surface

TLR4-functionalized surfaces were obtained following a previously reported protocol^23^. Briefly, carboxylate cuvettes were rinsed and equilibrated with PBS pH 7.4, and carboxylic groups were activated by EDC/NHS chemistry^40^. TLR4-His was solubilized in 10 mM CH_3_COONa, pH 4.5, then covalently coupled to the carboxylic surface *via* the N-terminus of the poly-His tail. To optimize surface density, different stock solutions of TLR4-His with concentrations in the range 200–1000 μg/mL were tested: 400 μg/mL was finally selected as it minimized steric hindrance, and at the same time prevented the dimerization between blocked TLR4-His macromolecules, both events being likely to reduce the number of available binding sites on the sensing surface. Free carboxylic sites on the sensor surface were inactivated by treatment with 1 M ethanolamine, pH 8.5. The surface was finally re-equilibrated with PBS.

The resulting shifts in the baseline (ΔR = 700–800 arcsec) generally indicated the assembly of a partial receptor monolayer for a 100 kDa protein (approximately 70% surface occupancy) corresponding to a final surface density of 1.20–1.30 ng/mm^2^, approximately equivalent to 9–10 mg/mL.

The use of CH_3_COONa 10 mM, pH 4.5 as immobilization buffer (chosen on the basis of TLR4-His isoelectric point 5.88) allowed an efficient immobilization. Negative baseline drift signals in TLR4 surface were not observed with time or upon multiple washes, confirming that the receptor molecules were irreversibly linked to the sensor surface.

### Determination of kinetic and equilibrium constants

Wheat ATI CM3 was added at six different concentrations in the range 16–600 nM to the TLR4 surface, each time monitoring association kinetics to equilibrium (approximately 2–4 min, depending on the concentration of ATI) prior to start dissociation phase.

Dissociation steps were performed with a single wash with PBS buffer, whereas baseline recovery was achieved by multiple washes with PBS supplemented with 200 mM NaCl, as ionic strength decreased the stability of the complex without affecting the functionality of the surface.

The determination of rate and equilibrium parameters was performed as described in detail elsewhere^41^. Briefly, upon addition of soluble ATI to surface-blocked TLR4, the amount of the ATI-TLR4 complex formed in time *t*, is given by:

1 $${[ATI-TLR4]}_{t}={[ATI-TLR4]}_{eq}[1-{e}^{-{k}_{on}t}]$$

where [*ATI* − *TLR4*]_*eq*_ is the concentration of complex at equilibrium. *k*_*on*_ is the concentration-dependent, pseudo-first order rate constant for the interaction where:

2 $${k}_{on}={k}_{ass}[ATI]+{k}_{diss}$$

The instrumental response is proportional to the mass of bound ligand, resulting in:

3 $${R}_{t}=({R}_{eq}-{R}_{0})[1-{e}^{-{k}_{on}t}]+\,{R}_{0}$$

where *R*_*t*_ is the response at time *t*, *R*_*0*_ is the initial response, and *R*_*eq*_ the maximal response at equilibrium for a given ligand concentration. From the fit of raw data, *k*_*on*_ values can be determined at any given concentration of ligand.

Hence, *k*_*ass*_ and *k*_*diss*_ were derived from the *k*_*on*_ vs ATI concentration plot. Equilibrium dissociation constant values were obtained both from the ratio of kinetic constants (*K*_*D*_ = *k*_*diss*_/*k*_*ass*_), and from the extent of the binding measured at equilibrium for any ligand concentration. In fact:

4 $${R}_{eq}={R}_{max}\frac{\frac{[ATI]}{{K}_{D}}}{1+\frac{[ATI]}{{K}_{D}}}$$

where *R*_*max*_ is the response at equilibrium obtained at asymptotic concentration of ligand. Once a complex has been formed, it will eventually dissociate into its components. Usually, the dissociation of surface-bound complexes may be described by:

5 $${R}_{t}=A{e}^{-{k}_{diss}t}$$

*A* is the extent of the dissociation phase. Binding analyses were repeated under different conditions to assess the influence of pH and ionic strength on the interaction (in the range of pH between 6 and 8, and salt concentration between 20 and 140 mM, respectively).

Raw binding data were analysed using the Fast Fit software (Fison Applied Sensor Technology) as previously reported^42^: the software uses an iterative curve-fitting to derive the observed rate constant and the maximum response at equilibrium due to ligand binding at a particular ligand concentration. Local and global fit analysis of the interaction data generally revealed monophasic kinetics. Specifically, mono-exponential analysis of association curves residuals was not affected by measurable systematic errors (a bi-exponential model did not significantly improve the quality of the fit as judged by an F-test, 95% confidence).

As a proof of specificity, wheat ATI produced non-significant signal upon addition to a bare carboxylate surface.

### Prediction of three-dimensional structure of wheat ATI

ATI CM3 precursor protein query sequence (P17314.1^43^) was obtained from UniProt database. The signal peptide (MACKSSCSLLLLAVLLSVLAAASA) was predicted using SignalP^44^, and the N-terminus sequence shortened accordingly. Fold-recognition was performed using I-Tasser^45^, the best structural templates being: 1B1U^46^, 4CVW^47^, 1BEA^48^, 1BFA^48^. The best predicted model had a TM-Score of 0.67 ± 0.13 (TM-score > 0.5 indicates a model of correct topology^49^). The model was refined and validated with Chiron-Gaia^50,51^.

### Protein-protein docking analysis

The predictive model of the complex between wheat ATI CM3 (UniProtKB ID: P17314) and human TLR4 was computed by docking ATI CM3 (obtained by fold-recognition) onto the crystallographic structure of the receptor (PDB ID: 3FXI^33^). Rigid docking was performed using PatchDock server^52,53^, ATI CM3 and TLR4 being uploaded as ligand and receptor, respectively, and FireDock^54,55^ was used for interaction refinement. Settings were always kept to default values. The best scoring complex and all images were rendered with PyMOL (The PyMOL Molecular Graphics System, Version 1.3 Schrödinger, LLC).

### Protein-peptide docking analysis

The most probable binding site for *RSGNVGESGLI* oligopeptide on human TLR4 (PDB ID: 3FXI^33^) was identified by flexible docking using the Autodock Vina software (version 1.1.2)^56^ on an Intel Core i7/Mac OSX 10.12-based platform. Hydrogen atoms were added to the receptor protein prior to any analysis. *RSGNVGESGLI* peptide was designed and energy minimized using Avogadro^57^ (Force field: MMFF94; Number of steps: 500; Algorithm: Conjugate gradients; Convergence: 10^−7^). Autodock Vina (a software performing a Lamarckian genetic algorithm to explore the binding possibilities of a ligand in a binding pocket^58^) was used with a grid of 76, 70, and 65 Å (in the x, y, and z directions) around the receptor, with a grid spacing of 0.375 Å, a root-mean-square (rms) tolerance of 0.8 Å, and a maximum of 2,500,000 energy evaluations. Other parameters were set to default values^59^. The obtained model has been further refined using NNScore^60^.

### Calculation of electrostatic potential maps

Electrostatic potential maps were determined using PDB2PQR server^61^ (including the APBS web solver), uploading the three-dimensional structures, and setting the parameters as default (Force field: PARSE). PROPKA was used for the determination of pKa.

### ATI digestion using pepsin under reducing conditions

ATI was treated in sequence with 2% 2-mercaptoethanol (10 min at 25 °C) to reduce intramolecular disulfide bonds, and with 14 mM iodoacetamide (30 min at 25 °C) to prevent re-oxidation of the thiols^62^. Resulting sample was extensively dialyzed against 20 mM sodium acetate, 150 mM NaCl, pH 4.0 to remove 2-mercaptoethanol and iodoacetamide, and eventually digested with pepsin according to the method described by Lin *et al*.^63^. Briefly, ATI was incubated with pepsin at 4 °C for 48 h, and the reaction was blocked by neutralization (pH = 7) with NaOH. The hydrolysis product was processed for binding to surface-blocked TLR4 as described above.

### Statistical analysis

Results were expressed as mean values ± standard deviation of results obtained from three independent experiments. Statistical analysis was performed with one-way ANOVA, followed by the Bonferroni test using Sigma-stat 3.1 software (SPSS, Chicago, IL, USA). *p* values < 0.01 were considered statistically significant.

## Electronic supplementary material

Supplementary Materials
