# Supplementary material for: Interfering with the high-affinity interaction between wheat amylase trypsin inhibitor CM3 and toll-like receptor 4: in silico and biosensor-based studies
Source: Sci Rep. 2017 Oct 13;7:13169. doi: 10.1038/s41598-017-13709-1 (PMC5640651; doi:10.1038/s41598-017-13709-1)
Supplement: Supplementary file 1 — Supplementary Materials [file 41598_2017_13709_MOESM1_ESM.pdf]

< Supplemental Material >

Interfering with the high-affinity interaction between wheat amylase trypsin inhibitor CM3 and toll-like receptor 4: *in silico* and biosensor-based studies

Massimiliano Cuccioloni[1,*], Matteo Mozzicafreddo[1], Laura Bonfili[1], Valentina Cecarini[1], Mara Giangrossi[1], Maurizio Falconi[1], Shin-Ichiroh Saitoh[2], Anna Maria Eleuteri[1], Mauro Angeletti[1].

[1]School of Biosciences and Biotechnology, University of Camerino (Italy)
[2]Division of Innate Immunity, Department of Microbiology and Immunology, The Institute of Medical Science, The University of Tokyo, 4-6-1 Shirokanedai, Minatoku, Tokyo 108 8639, Japan.

*CORRESPONDING AUTHOR

Massimiliano Cuccioloni

School of Biosciences and Veterinary Medicine – University of Camerino

Via Gentile III da Varano, 62032 Camerino MC - Italy

Email: massimiliano.cuccioloni@unicam.it

General materials

Tris, NaH_2PO_4, Na_2HPO_4, NaCl, HCl, NaOH and CH_3COONa were obtained from Mallinckrodt Baker (Milan, Italy). MES, DMSO, $MnCl_2$, $CaCl_2$, $CuSO_4$, $NiSO_4$, imidazole, KCl, Tween-20, 2-mercaptoethanol, iodoacetamide, chloroquine and DEAE dextran hydrochloride were obtained from Sigma-Aldrich (Milan, Italy). N-hydroxysuccinimide (NHS), 1-ethyl-3-(3-dimethylaminopropyl)-carbodiimide (EDC), ethanolamine and carboxylate cuvettes were obtained from Farfield Group (Cheshire, UK). All chemicals were of highest analytical grade. The plasmid pEF-BOS TLR4 expressing the extracellular domain of human TLR4 was produced as reported elsewhere[1]. Wheat samples were kindly provided by CERMIS (Centro Ricerche e Sperimentazione per il Miglioramento Vegetale "N. Strampelli" - Tolentino (MC), Italy). The Cary 1E UV-Vis spectrophotometer was obtained from Varian (Palo Alto, CA). The IAsys *Plus* biosensor was purchased from ThermoFisher Scientific (Milan, Italy). The AKTA chromatographic system equipped with a UV-Vis detector, HiTrap Metal-chelating columns were obtained from GE Healthcare (Milan, Italy), and a Tosoh Progel^{TM}-TSK G2000 SWXL column, 30 cm × 7.8 mm (Sigma Aldrich, Milan, Italy). Gel filtration standards were obtained from Bio-Rad Italia (Milan, Italy). The oligopeptide RSGNVGESGLI and the scrambled counterpart SGIVLSGGRNE were purchased from ProteoGenix SAS (Schiltgheim, France).

Expression and purification of TLR4

The plasmid pEF-BOS TLR4 was transformed into Top10 *Escherichia coli* competent cells prepared using calcium chloride. Bacteria harbouring pEF-BOS TLR4 were grown overnight at 37°C in 200 mL Luria-Bertani medium containing ampicillin at 60 µg/mL. The plasmid DNA was purified from this culture by the alkaline lysis method[2], and used to transfect HCT-116 cells. Plasmid transfection was performed as described by Frégeau and Bleackley[3]. Transfection procedure was optimized based on different experiments on the variations of plasmid, DEAE dextran and chloroquine concentrations, temperature and the duration of the extraction time. Briefly, HCT-116 cells were incubated at 37°C for 4 h in 10% FBS medium containing 1.0 µg/mL plasmid DNA and 100 µg/mL DEAE-dextran to allow plasmid uptake by cells *via* endocytosis. 100 µM chloroquine was included to inhibit degradation of plasmid DNA. After the incubation period, cells were exposed transiently to 10% DMSO to increase DNA uptake, and finally washed with PBS. Cell viability was examined with an inverted microscope. Transfected cells were pelleted, re-suspended in lysis buffer (20 mM Mops pH 7.0, 300 mM NaCl, 5 mM imidazole, 5% (v/v) glycerol, 10 mM β-mercaptoethanol, 0.3% (v/v) Triton X-100, 0.5 mM PMSF), and passed through a 25-gauge needle 10 times using a 1 mL syringe.

Cell homogenate was centrifuged at 15,000 × g for 30 min at 4°C, discarding the pellet. Finally, TLR4-His was purified by IMAC on a ÄKTA Basic chromatographic system using dedicated HiTrap Chelating HP columns charged with Cu^{2+}. The sample was applied at 1 column volume/min (CV/min) flow rate, and then the column was washed with 5 column volumes of binding buffer (0.02 M sodium phosphate, 0.5 M NaCl, 40 mM imidazole, pH 7.4) to remove non-tightly or non-specifically bound species. TLR4 was eluted using a linear gradient (0-100%, 4 CV) of elution buffer (0.02 M sodium phosphate, 0.5 M NaCl, 0.5 M imidazole, pH 7.4). The elution profile (Supplementary Fig.1, Panel A) showed three major peaks, the first corresponding to unbound species, the second to non-specifically retained species (eluted at low imidazole level), and the third corresponding to TLR4-containing fraction (eluted at approximately 7.5 min). TLR4 containing fraction was collected, dialyzed and stored at -20°C for further use. This fraction was separated by SE-HPLC with a TSK G2000SWXL column (isocratic elution with 0.1 M $NaSO_4$, 0.1 M NaH_2PO_4, pH 7; flow rate: 1 mL/min, λ: 280 nm). TLR4 was eluted at 7.3 min (Fig.1, Panel B). Protein purity (>95%) was assessed according to Papadoyannis and Gika[4]. Protein content was estimated using Coomassie blue-based reagent[5]. The purified product was freeze-dried and stored at -80°C.

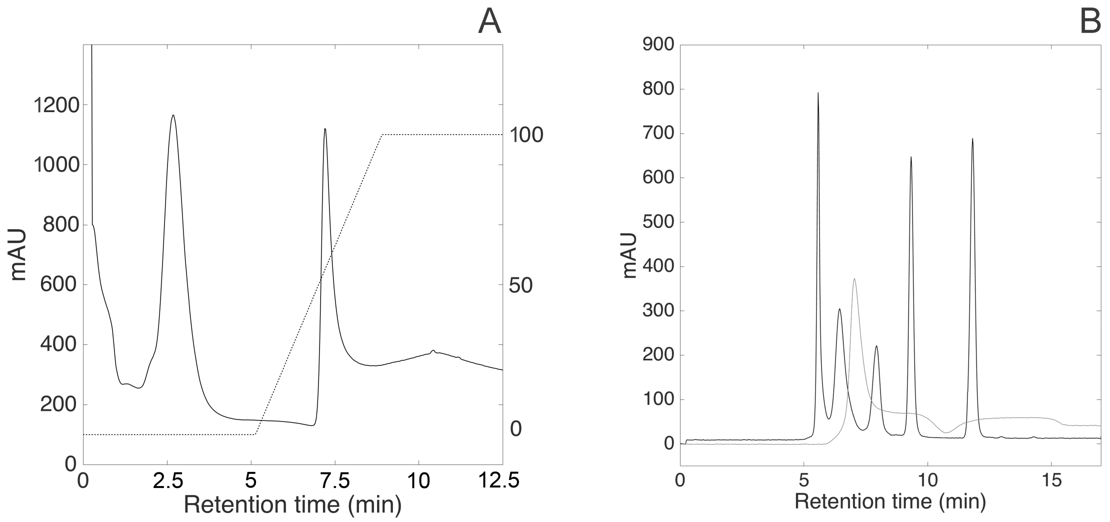

Supplementary Figure 1. Elution chromatogram of the lysate of HCT 116 cells transfected with pEF-BOS TLR4 plasmid DNA (Panel A): the peak at nearly 30 min corresponds to TLR4-containing fraction; dotted line indicates elution gradient. Gel filtration chromatogram of TLR4-containing peak (grey curve) superimposed to molecular weight markers (thyroglobulin, MW=670 kDa; γ-globulin, MW=158 kDa; ovalbumin, MW=44 kDa; myoglobin, MW=17 kDa; vitamin B12, MW=1.35) (Panel B).

Purification of ATI

Wheat ATI (CM3 variant) was isolated from commercial wheat samples as reported elsewhere[6]. Briefly, maize grains were crushed into fine powder and extracted in 50% (v/v) isopropanol for 30 min under gentle stirring at room temperature. Resulting suspension was centrifuged at 2,500 rpm for 15 min at room temperature to remove debris. Finally, ATI was purified from the supernatant using Cu^{2+}-charged HiTrap Chelating HP. Purified ATI was freeze-dried and stored at -80°C.

Electrostatic potential maps

A

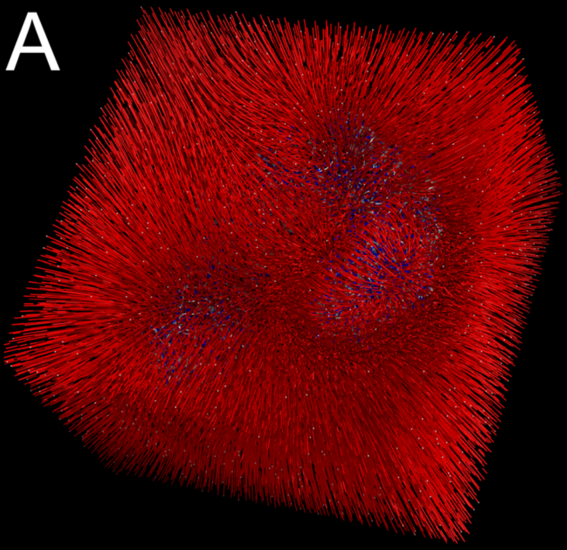

pH	Protein charge (pI = 5.56)
0.00	60.99
1.00	60.87
2.00	59.89
3.00	54.15
4.00	35.72
5.00	10.01
6.00	-5.83
7.00	-15.38
8.00	-19.37
9.00	-22.71
10.00	-32.52
11.00	-49.27
12.00	-64.09
13.00	-75.55
14.00	-80.72

B

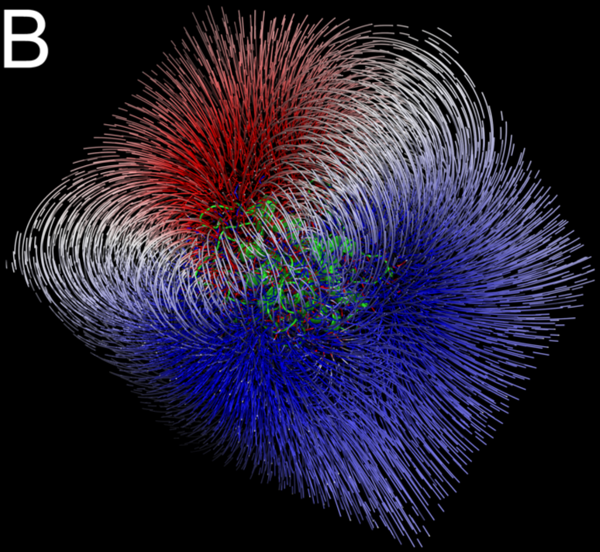

pH	Protein charge (pI = 7.48)
0.00	13.99
1.00	13.95
2.00	13.52
3.00	11.50
4.00	7.68
5.00	4.12
6.00	2.50
7.00	0.84
8.00	-0.71
9.00	-1.91
10.00	-4.73
11.00	-9.00
12.00	-12.85
13.00	-15.86
14.00	-17.55

Supplementary Figure 2. Electrostatic potential maps and dependence of protein charge from pH for human TLR4 (Panel A), and wheat ATI (Panel B).

Molecular docking of *RSGNVGESGLI* oligopeptide onto TLR4

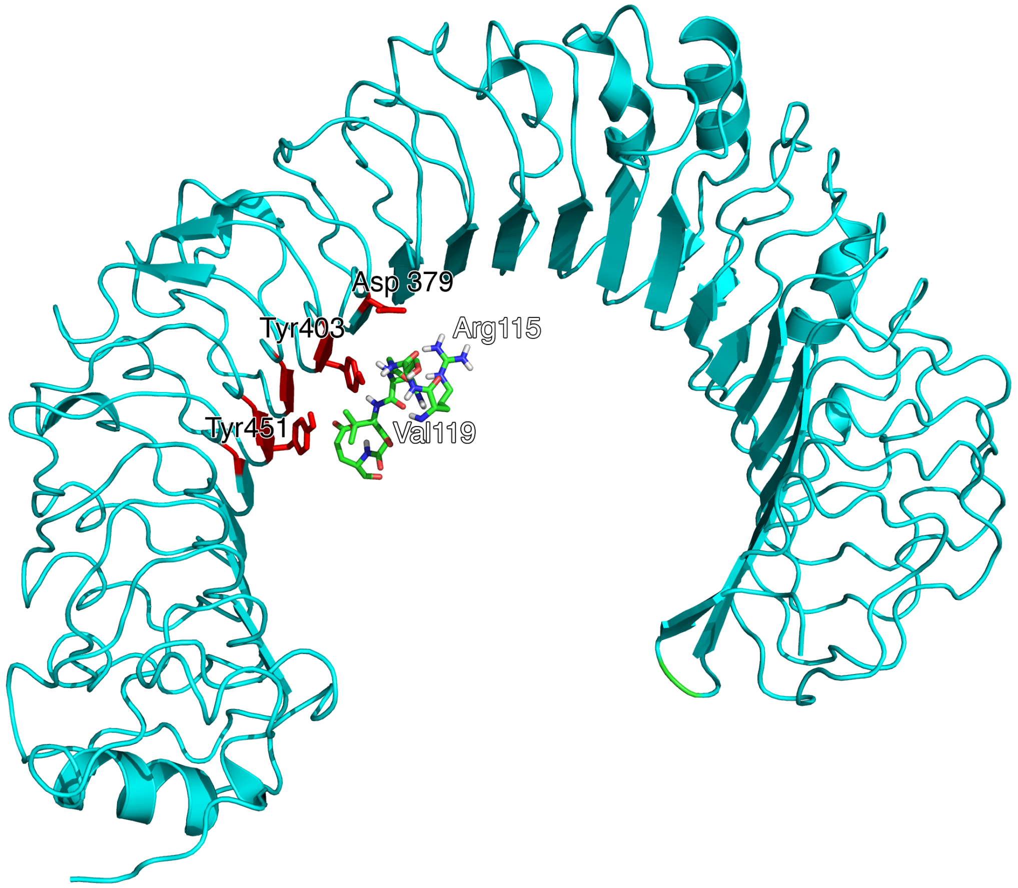

Supplementary Figure 3. Three-dimensional representation of molecular docking of RSGNVGESGLI peptide onto human TLR4 using Autodock Vina. The beta-sheets constituting the binding interface on TLR4 are highlighted in red, and all the residues involved in the formation of the complex are labelled. The complex was rendered with PyMOL software.

References

1. Shimazu, R.; Akashi, S.; Ogata, H.; Nagai, Y.; Fukudome, K.; Miyake, K.; Kimoto, M. MD-2, a molecule that confers lipopolysaccharide responsiveness on Toll-like receptor 4. *J Exp Med* **1999,** 189, 1777-82.
2. Sambrook, J.; Russell, D. W. *Molecular Cloning. A Laboratory Manual.* . CSHL Press: NY, 2001.
3. Fregeau, C. J.; Bleackley, R. C. Factors influencing transient expression in cytotoxic T cells following DEAE dextran-mediated gene transfer. *Somat Cell Mol Genet* **1991,** 17, 239-57.
4. Papadoyannis, I. N.; Gika, H. G. Peak purity determination with a diode array detector. *J. Liq. Chromatograph. Relat. Technol.* **2005**, 1083–1092.
5. Bradford, M. M. A rapid and sensitive method for the quantitation of microgram quantities of protein utilizing the principle of protein-dye binding. *Anal Biochem* **1976,** 72, 248-54.
6. Cuccioloni, M.; Mozzicafreddo, M.; Ali, I.; Bonfili, L.; Cecarini, V.; Eleuteri, A. M.; Angeletti, M. Interaction between wheat alpha-amylase/trypsin bi-functional inhibitor and mammalian digestive enzymes: Kinetic, equilibrium and structural characterization of binding. *Food Chem* **2016,** 213, 571-8.
